# International Survey on Evidence for Index Lymph Node Surgery After Neoadjuvant Systemic Therapy for Stage III Melanoma

**DOI:** 10.1245/s10434-025-18475-3

**Published:** 2025-10-14

**Authors:** Elan Novis, Mervi Rautalin, Rodabe N. Amaria, Paolo A. Ascierto, Christian U. Blank, Mark B. Faries, Dirk J. Grunhagen, David E. Gyorki, Andrew J. Hayes, Anke M. J. Kuijpers, Georgina V. Long, Joshua M. V. Mammen, Alexander M. Menzies, Merrick I. Ross, Piotr Rutkowski, Hussein A. Tawbi, Michael T. Tetzlaff, Jonathan S. Zager, Jennifer A. Wargo, Tina J. Hieken, Alexander C. J. van Akkooi, and on behalf of The International Neoadjuvant Melanoma Consortium (INMC)

**Affiliations:** 1 Melanoma Institute Australia, Sydney, NSW Australia; 2 Faculty of Medicine and Health, University of Sydney, Sydney, NSW Australia; 3 Department of Melanoma and Surgical Oncology, Institute of Academic Surgery, Royal Prince Alfred Hospital, Sydney, NSW Australia; 4 The Royal Marsden Hospital, London, UK; 5 Department of Plastic Surgery, Helsinki University Hospital and University of Helsinki, Helsinki, Finland; 6 Department of Melanoma Medical Oncology, UT MD Anderson Cancer Center, Houston, TX USA; 7 Istituto Nazionale Tumori IRCCS Fondazione G. Pascale, Naples, Italy; 8 The Netherlands Cancer Institute, Amsterdam, The Netherlands; 9 The Angeles Clinic and Research Institute, A Cedars-Sinai Affiliate, Los Angeles, CA USA; 10 Erasmus MC, Rotterdam, The Netherlands; 11 Division of Cancer Surgery, Peter MacCallum Cancer Centre and Sir Peter MacCallum Department of Oncology, University of Melbourne, Melbourne, VIC Australia; 12 Royal North Shore and Mater Hospitals, Sydney, NSW Australia; 13 Department of Surgery, University of Nebraska Medical Center, Omaha, NE USA; 14 Department of Surgical Oncology, UT MD Anderson Cancer Center, Houston, TX USA; 15 Maria Sklodowska-Curie National Research Institute of Oncology, Warsaw, Poland; 16 Departments of Pathology and Dermatology; Dermatopathology and Oral Pathology Unit, University of California San Francisco, San Francisco, CA USA; 17 Department of Cutaneous Oncology, Moffitt Cancer Center, Tampa, FL USA; 18 Department of Oncologic Sciences, University of South Florida Morsani College of Medicine, Tampa, FL USA; 19 Mayo Clinic Comprehensive Cancer Center, Rochester, MN USA

**Keywords:** Melanoma, Neoadjuvant, Index lymph node, Stage III

## Abstract

**Background:**

Neoadjuvant immunotherapy for resectable stage III melanoma has demonstrated promising outcomes in recent trials, prompting a change in clinical practice in many countries. Although therapeutic lymph node dissection (TLND) remains the standard of care after neoadjuvant treatment, a less invasive index lymph node (ILN)-guided approach has been proposed. The global melanoma community’s acceptance of neoadjuvant immunotherapy and the need for TLND or ILN after this remains unclear.

**Methods:**

A two-stage international survey was conducted among melanoma experts between May 2023 and January 2025. Respondents were asked about their familiarity with neoadjuvant trials, current practices, and opinions on ILN versus TLND before and after publication of the NADINA trial.

**Results:**

The response rates were 50% (118/237) in the first survey and 62% (148/237) in the second survey. In the second survey, 74% of the respondents considered neoadjuvant therapy the standard of care, and support for ILN-guided surgery rose from 27 to 40% between the surveys. However, 54% still favored a phase 3 randomized controlled trial before changing the clinical practice guidelines, and only 27% believed the current data were sufficient for adoption of ILN as standard. Key barriers included concerns about oncologic safety, pathologic standardization, and patient selection.

**Conclusion:**

The current evidence supports the use of neoadjuvant immunotherapy as the standard of care for stage III melanoma. However, widespread clinical adoption of ILN-guided surgical de-escalation remains limited. A multicenter phase 3 trial (MSLT-3), launching in 2025, is expected to provide important data to guide future practice.

**Supplementary Information:**

The online version contains supplementary material available at 10.1245/s10434-025-18475-3.

Macroscopic stage III melanoma is an aggressive cancer. The 5-year melanoma-specific survival is 83% for stage IIIB melanoma, but decreases together with staging, from 69% for stage IIIC to a mere 32% for stage IIID melanoma.^1^

Recent large studies have examined the role of neoadjuvant treatment for this patient group, with results favoring administration of neoadjuvant treatment over conventional adjuvant systemic therapy after surgery.^2,3^ The randomized phase 2 SWOG S1801 trial compared therapeutic lymph node dissection (TLND) followed by pembrolizumab (fixed dose of 200 mg Q3W for a maximum of 18 doses) with a perioperative regimen of pembrolizumab (3 doses before surgery followed by TLND and up to 15 doses after surgery). They found a 23% absolute benefit in event-free survival (EFS) at 2 years for the perioperative regimen versus the adjuvant treatment (72% vs. 59%).^2^ Similarly, the randomized phase 3 NADINA trial compared TLND plus adjuvant nivolumab (480 mg Q4W for a maximum of 12 doses) with neoadjuvant ipilimumab (80 mg Q3W) plus nivolumab (240 mg Q3W) for 2 doses) followed by TLND and response-driven adjuvant therapy after surgery. This study demonstrated an absolute benefit of EFS of 24.8% (84.5% vs. 59.7%) at 1 year.^3^

Results from these trials have forged a shift in the treatment paradigm to neoadjuvant immunotherapy for macroscopic stage III melanoma as the new standard of care.^4–6^ In these seminal studies, patients were treated with TLND for stage III melanoma, which is the standard of care.

It is well-known that TLND is associated with significant short- and long-term morbidity.^7^ Common short-term complications include surgical-site infections, seromas, drain complications, and wound breakdown. These can occur in approximately 40% of patients, particularly after inguinal lymphadenectomy.^7,8^ The most important long-term complication is chronic lymphoedema of the affected limb, with rates between 20 and 35% for the axilla and reaching 64% for the groin.^7–9^ It is well known that lymphedema is associated with a significant deterioration in quality of life for the affected individuals.^10^

A reassessment of the pathologic response of patients enrolled in Optimal Adjuvant Combination Scheme of Ipilimumab and Nivolumab in Melanoma Patients (OpACIN) and Optimal Neo-Adjuvant Combination Scheme of Ipilimumab and Nivolumab (OpACIN-neo) demonstrated that the pathologic response assessment of only the index lymph node (ILN) was a reasonable surrogate of the response assessment of the entire TLND specimen.^11^

The PRADO trial next examined ILN surgery as an alternative treatment strategy.^12^ The ILN was defined as either the only involved lymph node at baseline or the largest metastatic lymph node (in the case of multiple involved lymph nodes). The ILN was selectively removed and examined after neoadjuvant treatment. To be able to accurately retrieve the lymph node, it was clipped at baseline^13^ before the initiation of neo-adjuvant immunotherapy.

The results of the pathologic response assessment informed subsequent treatment decisions.^12,14,15^ For patients with a major pathologic response (MPR, defined as pathologic complete response [pCR] or near-pCR [max 10% viable tumor cells]) in the ILN, TLND was not performed. Because 61% of the patients achieved an MPR to the neoadjuvant immunotherapy (in this case, ×2 ipilimumab 1 mg/kg and nivolumab 3 mg/kg Q3W), only 30 of 99 patients underwent a TLND as part of their initial disease management.^12^ Only 4 of the 60 patients for whom direct TLND was omitted had a recurrence during a follow-up time of 28.1 months. Three of these four recurrences were at the lymph node basin and could be managed with a delayed TLNDs.^12^ The remaining nine patients experienced toxicity or disease progression and did not proceed with ILN assessment.

The European Society for Medical Oncology (ESMO) recently published updated guidelines for cutaneous melanoma recommending that neoadjuvant treatment be offered to patients with resectable macroscopic stage III melanoma, followed by surgical treatment, but the extent of surgical treatment was not specified. In case of major pathologic response, adjuvant treatment should be omitted.^4^

Recently, the International Neoadjuvant Melanoma Consortium (INMC) discussed the current evidence of neoadjuvant treatment for stage III melanoma patients, including difficulties of radiologic assessment including the possibility of disease progression during reception of neoadjuvant treatment and implementation of the ILN approach.^16,17^ The INMC consensus was that PRADO, although yielding exciting results, was too small (*n* = 99) without mature follow-up data and should therefore be considered a proof-of-concept study.

Whether to change the standard of care to ILN surgery or to continue with TLND after neoadjuvant treatment remains a critical unanswered question. Moreover, the potential pathway toward implementation of an ILN approach after neoadjuvant immunotherapy and what additional data might be required also are points of discussion. Therefore, to examine the opinions of melanoma experts worldwide, we designed a survey to evaluate current opinions on the role of neoadjuvant immunotherapy for resectable stage III melanoma. We also studied what the next steps would be in the evaluation of the role and implementation of ILN surgery after neoadjuvant treatment.

## Material and Methods

**Fig. 1:**
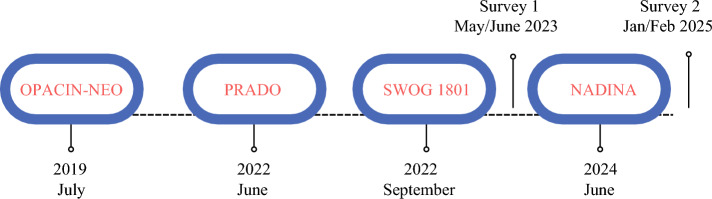
Timeline of surveys in relation to key clinical trials.

The Melanoma-Index Lymph Node (ILN) Survey was designed and tested first with six local melanoma experts. Then, after revision of the questionnaire, it was further tested with three international experts and two fellows. After subsequent final adjustments, the survey was sent on 25 May 2023 to 215 international melanoma experts through different platforms (Fig. 1). The survey was created using SurveyMonkey, a web-based questionnaire platform. The second round of invitations was sent out on 1 June 2023, reaching 22 additional melanoma clinicians.

A second follow-up questionnaire was created in similar fashion and circulated to the same group of clinicians on 29 January 2025 after the results of the NADINA trial were published to gauge whether this had caused a change in practice across melanoma centers. Automatic reminders were sent 2 weeks later to those who did not reply to the initial invitation or had incomplete responses. The final questions and potential answers for each survey are summarized in Tables S1 and S2.

The questionnaire comprised questions of personal demographics (age, gender, clinical experience and specialty, country of origin) and specific questions of knowledge about current research results regarding neoadjuvant treatment and ILN versus TLND, opinions on the validity of available data in reflection of clinical practice guidelines and standard of care, and details of the respondents’ current practices. Additional information was provided in the survey about study results such as the OpACIN-Neo study, PRADO and SWOG 1801 results. Supplemental material number 1 summarizes all the questions. Respondents’ background factors (i.e., age, gender, specialty, or country of origin) were considered as expert opinions regardless of demographic factors and thus are presented as percentages of the answers received.

## Results

**Fig. 2:**
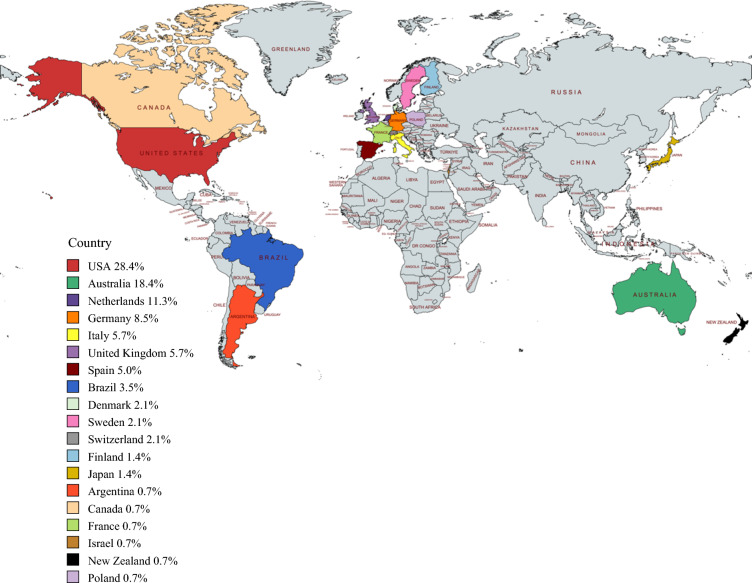
Percentage of respondents by country.

The response rates were 50% (118/237) for the first survey and 62% (148/237) for the second survey. The responses to the first survey were received from the United States (29%), The Netherlands (22%), Australia (14%), Germany (8%), UK (4%), and other countries (23%). The majority of the responses to the second survey were from the United States (28%), Australia (18%), and The Netherlands (11%). A summary of the respondents’ demographics are presented in Table 1, and country distributions are shown in Fig. 2.

**Table 1:** Respondent demographic factors

Characteristic	Survey 1 (*n* = 118) *n* (%)	Survey 2 (*n* = 148) *n* (%)
*Gender*		
Female	42 (36)	43 (29)
Male	76 (64)	104 (70)
Other	0 (0)	1 (1)
*Specialty*		
Dermatology	13 (11)	21 (14)
Medical oncology	31 (26)	47 (32)
Surgery	72 (61)	69 (47)
Other	2 (2)	11 (7)
*Age (years)*		
25–34	6 (5)	6 (4)
35–44	43 (36)	31 (21)
45–54	38 (32)	59 (40)
55+	32 (27)	52 (35)
*Years board-certified*		
0–5	17 (14)	12 (8)
6–10	21 (18)	19 (13)
11–15	28 (24)	34 (23)
16–20	13 (11)	25 (17)
21–25	11 (9)	16 (11)
>25	25 (21)	37 (25)
Not yet certified	3 (3)	5 (3)

### Evidence for Neoadjuvant Immunotherapy for Resectable Stage III Disease

The majority of the respondents in the initial survey were largely familiar (37% extremely familiar and 49% very familiar) with previous neoadjuvant studies of melanoma (e.g., OpACIN-neo, SWOG-1801, and PRADO). In the second survey, the respondents were also largely familiar with the results of the NADINA trial, with 89% stating they were either very or extremely familiar with the results of this trial. Although 96% of the respondents in the first survey believed that neoadjuvant immunotherapy for macroscopic stage III melanoma will soon become the new standard of care based on the current evidence, 74% of the respondents in the second survey believed that this is already considered the standard of care based on the NADINA trial results and other available evidence.

Pembrolizumab alone (24%) or ipilimumab plus nivolumab (25%) was the most common drug used in the neoadjuvant setting, whereas 36% of those surveyed stated that a tailored approach to drug therapy is adopted by their institution based, for example, on the patient’s comorbidities.

### Evidence for Index Lymph Node Response-Driven Treatment

**Fig. 3:**
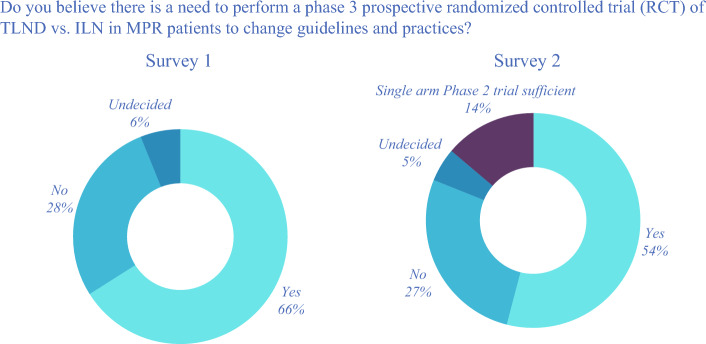
Pie chart demonstrating change in responses across two surveys.

In the first survey, 49% of the respondents either strongly agreed or agreed that the current evidence was sufficient to change management guidelines in support of de-escalating surgery using ILN response-driven treatment rather than TLND. Although 31% disagreed and 19% remained undecided, 66% of the respondents believed that a phase 3 prospective randomized control trial is needed to change guidelines and current practice (Fig. 3).

In the second survey, still 54% of the respondents believed a phase 3 randomized controlled trial (RCT) is needed to support ILN-guided treatment to become the standard of care. Likewise, 60% of the respondents did not believe that a single-arm phase 2 trial comparing ILN and “historical” TLND results after MPR would be sufficient to change practice. After publication of the NADINA trial, 27% of the respondents in the second survey believed that existing data were sufficient to change current practice and guidelines, compared with 6% of the respondents in the initial survey.

### Trial Design for a Phase 3 Randomized Controlled Trial

In terms of endpoints for a prospective trial, the majority of the responses (44%) suggested relapse-free survival (RFS) or event-free survival (EFS) as the most appropriate primary endpoint. However, 86% of the respondents believed they could recruit fewer than 20 patients to a randomized phase 3 trial annually. Whereas 55% of the respondents would opt for a single-agent anti-PD1 therapy alone as part of a phase 3 trial protocol, 29% suggested they would participate only if ipilimumab and nivolumab were the drugs of choice for the trial.

Only 27% of the centers in the first survey currently offered ILN surgery or planned to offer it in the next 1 or 2 years even without additional trial data. In the second survey, this increased to 40% of the respondents, with 26% stating they would adopt a selective ILN approach based on location, number of involved nodes, or radiologic response. The preferred methods of ILN identification were metallic clip insertion (30%), magnetic seed (17%), and radioactive seed (14%). However, most of the centers surveyed did not currently offer ILN surgery outside of trials (56% in the first survey, 53% in the second survey).

### Free Comments

In the free comment sections, the respondents were asked what they thought were the greatest barriers to ILN becoming the standard of care. The most common themes were pathologic standardization of response evaluation, evidence of oncologic safety, and issues with patient selection (i.e., delay of definitive care for patients who do not have MPR). The respondents also had some concern regarding transition to unresectable disease with staged surgery. Some respondents raised concerns around the ability to enroll sufficient numbers to achieve statistical power for a phase 3 trial. In addition, comments regarding a potential lack of equipoise were raised, such as scenarios in which patients who have MPR might refuse to proceed to TLND or clinicians’ bias and reluctance to enroll patients in these trials because of a desire to avoid a TLND.

## Discussion

This study highlighted the opinions and current clinical practice of the global melanoma multidisciplinary community in treating patients with resectable stage III melanoma. Given the rapidly changing evidence in this field during recent years, it is important to identify gaps in current knowledge to help design prospective trials that will be clinically relevant and robust.

Through the evolution of these two surveys, the initial one before the publication of the NADINA trial^3^ based on evidence from SWOG-1801, OpACIN-neo, and PRADO,^2,12,18^ and the second one that incorporated knowledge of the NADINA trial results, we have witnessed a shift in the opinions of the worldwide melanoma community in favor of neoadjuvant therapy and implementation of ILN-guided treatment for stage III melanoma. Since the Nadina trial, nearly three fourths of respondents believe that neoadjuvant immunotherapy currently is considered the standard of care in this setting. Although more respondents believed ILN-guided treatment currently was the standard of care in the second survey, still, 55% of the respondents continue to perform TLND as the surgical approach after neoadjuvant immunotherapy. Moreover, 68% of the respondents believe that further evidence is required, including 54% who believe a phase 3 trial is needed. This demonstrates the need for a well-designed, multicenter phase 3 trial to help answer this question.

**Fig. 4:**
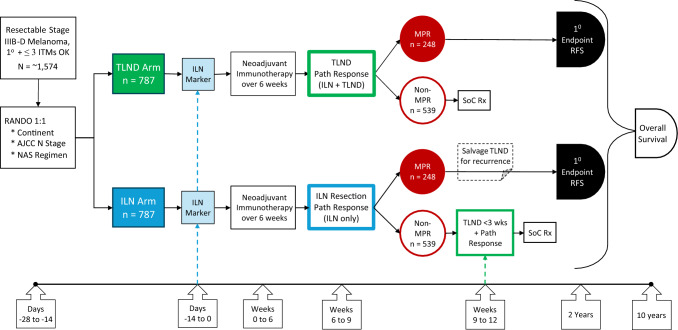
Design of Multicenter Selective Lymphadenectomy Trial-3 (MSLT-3).

In proposing the Multicenter Selective Lymphadenectomy Trial-3 (MSLT-3; Fig. 4), the majority of the respondents believed this study would provide adequate evidence to answer this question (84%), and importantly, the majority of the respondents would be willing to contribute to the trial (74%). Those who suggested that they would not participate in a potential randomized trial argued that not enough clinical equipoise existed to randomize their patients to the TLND group because they believed enough evidence currently exists to suggest that an ILN could be performed safely. In the absence of any high-quality data to confirm this apart from the PRADO study, which was a single proof-of-concept trial of 99 patients only and not considered the standard of care, we believe that a larger RCT is needed to prove non-inferiority. The majority of the respondents were in agreement with this, with only 27% of the respondents arguing that currently, enough evidence was available already to change practice.

An important aspect of any prospective trial evaluating the feasibility of ILN-guided treatment is to be certain that centers adopting this technique are accurately identifying the true index node. The index lymph node should be marked before the commencement of neoadjuvant therapy given that up to two thirds of nodes will become non-palpable after treatment.^19^ We therefore thought it would be helpful to determine what techniques are being used around the world to help determine which techniques could be included in future trial designs. The most common techniques used for ILN included metallic clips, magnetic or radioactive seeds, and radar guided localization. These techniques have been validated as a surgical technique used for identification of index/involved lymph nodes that may decrease in size after neoadjuvant therapy and become non-palpable. Therefore, any future studies should ensure that at least one technique is used to confirm that the correct node is being resected to help guide further treatment decisions.^13,19^

The limitations of this survey included the variation in all aspects of melanoma treatment worldwide, including surgical approaches, access to and reimbursement of immunotherapy drugs, imaging services, and the like. Likewise, the respondents may have expressed their own personal beliefs rather than the standard practice at their institutions. Another limitation of the survey was that the respondents requested to participate all were contacts known to the survey designer rather than respondents from a group of society member or networks, potentially presenting a biased view from tertiary centers rather than community practices. Finally, the inherent contraindication in the survey results, with 49% of the respondents believing that evidence was sufficient to change practice, yet with 66% of respondents in the same questionnaire considering an existing need for a phase 3 RCT, suggests some ambivalence and lack of confidence to implement change.

Nevertheless, this survey likely reflects the trends and opinions of melanoma experts globally, with a good spread of responses across five continents. Moreover, community practice tends to follow expert tertiary practice. To abolish the standard practice of TLND worldwide on the largest scale, including community practice, we believe a phase 3 RCT will be most impactful. Despite half of the patients on the MSLT-3 trial requiring a TLND, it might prevent unnecessary TLND and associated surgical morbidity for a larger number of patients.

This survey clearly showed that the melanoma community is adopting the findings of the neoadjuvant trials and that the majority currently consider neoadjuvant therapy the standard of care. However, further evidence is needed for adoption of the ILN technique as first proposed in the PRADO study. We look forward to conducting the phase 3 multicenter randomized MSLT-3 trial, which will begin during September 2025 in Australia and is expected to reach full recruitment (including sites in the United States, Canada, Brazil, United Kingdom, EU, Israel, Japan, and New Zealand) by 2028 or 2029, with the primary endpoint presented by 2030 or 2031.

## Conclusion

This survey gives insight into the views of the international multidisciplinary melanoma medical community regarding the role of neoadjuvant immunotherapy as well as how to proceed to implement surgical de-escalation after immunotherapy. A phase 3 RCT was the preferred option. The survey demonstrated that with increased time, experience increased and opinions started to shift, but the majority still performed TLND and preferred a phase 3 RCT. An RCT (MSLT3) is scheduled to enroll the first patient in 2025.

## Supplementary Information

Below is the link to the electronic supplementary material.

Supplementary file1 (DOCX 23 KB)
