# Supplementary material for: International Survey on Evidence for Index Lymph Node Surgery After Neoadjuvant Systemic Therapy for Stage III Melanoma
Source: Ann Surg Oncol. 2025 Oct 14;33(3):2571–8. doi: 10.1245/s10434-025-18475-3 (PMC12901128; doi:10.1245/s10434-025-18475-3)
Supplement: Supplementary file 1 — Supplementary file1 (DOCX 23 KB) [file 10434_2025_18475_MOESM1_ESM.docx]

**Supplementary Table 1: Survey 1 questions and answer options**

|  | Question Text | Answer Options |
| --- | --- | --- |
| Q1 | We would kindly like to ask you consent to use your answers in this survey for a research study… Do you consent to this? | Yes; No |
| Q2 | What is your age? | 18–24; 25–34; 35–44; 45–54; 55–64; 65–74; 75 or older |
| Q3 | What is your medical specialty? | (General) Surgery; Surgical Oncology; Plastic Surgery; Head & Neck Surgery; Dermatology; Dermato‑Oncology; Dermato‑Surgery; Medical Oncology; Radiation Oncology; Other |
| Q4 | What is your gender? | Female; Male; Other |
| Q5 | How long have you been a board certified medical specialist? | 0–5 years; 6–10; 11–15; 16–20; 21–25; >25; Not yet board certified / fellow; Other |
| Q6 | In what country do you work? | [List of all countries; respondents selected one] |
| Q7 | Are you familiar with the results of the neo‑adjuvant immunotherapy studies in melanoma (for example: OpACIN‑neo, SWOG‑1801 and PRADO)? | Extremely familiar; Very familiar; Somewhat familiar; Not so familiar; Other |
| Q8 | Based on the presented and published results so far (for example; OpACIN‑neo, PRADO, SWOG‑1801), do you believe neo‑adjuvant immunotherapy will soon become the new standard of care treatment for macroscopic stage III melanoma? | Very likely; Likely; Neither likely nor unlikely; Unlikely; Very unlikely; Other |
| Q9 | How much do you agree with the following statement: The current body of evidence is already sufficient to support changing guidelines and surgical practice towards a de‑escalation approach using the ILN and avoiding TLND in patients who have achieved an MPR on neo‑adjuvant immunotherapy. | Strongly agree; Agree; Neither agree nor disagree; Disagree; Strongly disagree; Other |
| Q10 | Do you believe there is a need to perform a phase 3 prospective randomized controlled trial (RCT) of TLND vs. ILN in MPR patients to change guidelines and practices? | Yes; No; Other |
| Q11 | Do you think a single‑arm prospective phase 2 trial that compares ILN versus 'historical' TLND results after MPR patients is sufficient to change guidelines and surgical practices? | Yes; No; Other |
| Q12 | If we would perform any type of prospective trial, what would be the most appropriate primary endpoint for you? | Relapse‑Free Survival (RFS) / Event‑Free Survival; Distant Metastasis‑Free Survival; Regional Control Rate; Overall Survival; Quality of Life; Other |
| Q13 | If you had to rank… the following options to make ILN a novel standard of care: No trial / Phase 3 RCT / Single‑arm Phase 2 — how would you rank these scenarios? | (Ranking scale for 3 options) |
| Q14 | Do you offer an ILN outside of trials currently or do you plan to do so within the next 12–24 months (without any new additional trial data to support that)? | Yes; No; Other |
| Q15 | If you offer ILN already now, in which scenarios do you consider using an ILN approach? (multiple options possible) | Do not yet offer ILN; Single involved node at baseline; Depending on lymph node basin; Depending on radiological/metabolic response; Other |
| Q16 | Considering all existing evidence and the statistical power of the different scenarios, does this change your opinion on how we should proceed to establish the ILN as a novel standard of care for macroscopic stage III melanoma? | No, existing data sufficient; No, support single‑arm Phase 2; No, support Phase 3 RCT; Yes, existing data is sufficient; Yes, need single‑arm Phase 2; Yes, need Phase 3 RCT; Other |
| Q17 | How many patients would you anticipate to be able to recruit to a phase 3 RCT per annum? | 0–5; 5–10; 10–20; 20–50; 50+; None |
| Q18 | How many patients would you anticipate to be able to recruit to a single‑arm prospective phase 2 per annum? | 0–5; 5–10; 10–20; 20–50; 50+; None |
| Q19 | Would the choice of drug(s) available for the study influence your decision? | Yes, only if IPI/NIVO; No, single-agent anti‑PD‑1 is sufficient; Other |
| Q20 | Please share your view about what is needed to make sure the ILN approach becomes the new standard of care for MPR patients. | (Open‑text) |
| Q21 | What is the biggest barrier to making the ILN the novel standard of care? | (Open‑text) |
| Q22 | What problems do you foresee with either trial design? | (Open‑text) |
| Q23 | Final comments? | (Open‑text) |

**Supplementary Table 2: Survey 2 questions and answer options**

|  | Question Text | Answer Options |
| --- | --- | --- |
| Q1 | We would kindly like to ask you consent to use your answers in this survey for a research study… Do you consent to this? | Yes; No |
| Q2 | What is your age? | 18–24; 25–34; 35–44; 45–54; 55–64; 65–74; 75 or older |
| Q3 | What is your medical specialty? | (General) Surgery; Surgical Oncology; Plastic Surgery; Head & Neck Surgery; Dermatology; Dermato‑Oncology; Dermato‑Surgery; Medical Oncology; Radiation Oncology; Other |
| Q4 | What is your gender? | Female; Male; Other |
| Q5 | How long have you been a board certified medical specialist? | 0–5 years; 6–10; 11–15; 16–20; 21–25; >25; Not yet board certified / fellow; Other |
| Q6 | In what country do you work? | [List of all countries; respondents selected one] |
| Q7 | Are you familiar with the results of the NADINA Trial (Blank et al. 2024 NEJM)? | Extremely familiar; Very familiar; Somewhat familiar; Not so familiar; Other |
| Q8 | Based on presented and published results so far (including NADINA; SWOG‑1801, INMC pooled analyses and PRADO), do you believe neo‑adjuvant immunotherapy will soon become, or is already considered, standard for macroscopic stage III melanoma? | Already considered standard of care; Soon to become standard but further evidence needed; Undecided; Unlikely; Other |
| Q9 | What regimen of immunotherapy is currently being used at your institution for neo‑adjuvant therapy of macroscopic stage III melanoma (non‑clinical trial)? | Pembrolizumab only; Nivolumab only; Ipilimumab + Nivolumab; Nivolumab + Relatlimab; Combination depending on comorbidities; N/A; Other |
| Q10 | What is the current surgical treatment protocol at your institution following neoadjuvant immunotherapy for macroscopic stage III melanoma? | TLND for all patients; ILN followed by TLND based on pathological response; Selective ILN; Other |
| Q11 | If performing, or planning to perform ILN resection, what method are you using to identify the index node? | N/A; Magnetic seed; Metallic clip; Radar‑guided localization or ROLL; Radioactive seed; Wire; Other |
| Q12 | What evidence do you think is required in order for ILN to become the standard of care in macroscopic stage III melanoma? | Phase 3 Randomized Controlled Trial (RCT); Single‑arm Phase 2 trial; I believe existing data is sufficient; Other |
| Q13 | Do you think the above trial design will provide sufficient evidence to support ILN as the standard of care…? | Yes; No – please specify reason below |
| Q14 | Do you think these outcomes are appropriate to answer the clinical question in demonstrating non‑inferiority of ILN followed by no further treatment for MPR patients, compared to TLND? | Yes; No – a different primary outcome should be assessed; No – additional secondary outcome should be assessed |
| Q15 | Would you be willing to participate in this Phase 3 randomized trial? | Yes; No; Undecided |
| Q16 | How many patients would you anticipate to be able to recruit to a phase 3 RCT per annum? | None; 1–5; 6–10; 11–20; 21–50; 51 or more |
| Q17 | What problems do you foresee with this trial design? | (Open‑text) |
| Q18 | Final comments? | (Open‑text) |
